# Microbial Transglutaminase—The Food Additive, a Potential Inducing Factor in Primary Biliary Cholangitis

**DOI:** 10.3390/molecules30040762

**Published:** 2025-02-07

**Authors:** Alicja Bauer, Paulina Rosiek, Tomasz Bauer

**Affiliations:** 1 Department of Biochemistry and Molecular Biology, Centre of Postgraduate Medical Education, Marymoncka 99/103, PL-01-813 Warsaw, Poland; alicja.bauer@cmkp.edu.pl; 2 Faculty of Chemistry, University of Warsaw, L Pasteura 1, PL-02-093 Warsaw, Poland; pe.morawska2@student.uw.edu.pl

**Keywords:** microbial transglutaminase, primary biliary cirrhosis, molecular mimicry, autoimmunity

## Abstract

Microbial transglutaminase (mTG) is a bacterial survival factor, which is frequently used as a food additive. This results in the formation of immunogenic epitopes that may cause autoimmunity. Primary biliary cholangitis (PBC) is a cholestatic, autoimmune liver disease characterized by the presence of characteristic autoantibodies. The aim of this work was to determine epitope similarity and cross-reactivity between mTG- and PBC-specific antigens and to investigate whether the microbial enzyme may be associated with the induction of autoimmunity due to epitope similarity and cross-reactivity. Monoclonal and polyclonal antibodies against mTG were applied to nine different PBC-specific antigens using ELISA technique. They reacted significantly with four out of nine antigens. This reaction was most pronounced for gp210 and PML protein. We also performed in vitro studies on the impact of the mTG on the specific antigen–antibody binding using sera of PBC patients. We found four PBC-specific antigens that share homology with mTG sequences. We noticed inhibition of this specific binding by the mTG to the PDC M2, gp210, PML, and KLHL12 protein. Microbial mimics may be the major targets of cross-reactivity with human-specific antigens. Cross-reactivity may indicate a link between mTG and the development of autoimmune diseases.

## 1. Introduction

Microbial TG, an extracellular enzyme of the class of transferases, is produced commercially through traditional fermentation by the microorganism *Streptoverticillium moboarense* [1]. It is a monomer consisting of 331 amino acids and is almost half the size of transglutaminase of animal origin. Its molecular mass is 37,900 Da [1,2]. Transglutaminase catalyzes the transport of acyl groups, protein deamination, and polymerization of inter- and intramolecular proteins. Reactions catalyzed by mTG allow changes in the properties of proteins present in the food matrix [3]. Microbial TG also enhances flavor, improves appearance, and enables the creation of new processed food products.

It is widely known to modify protein functional properties in food [1,2,3,4]. An assortment of food proteins were described as substrates for mTG: milk and whey proteins, soy globulins, myofibrillar proteins, albumins, and others [5,6].

Recently, there have been reports regarding microbial transglutaminase (mTG) as a potential factor that may induce autoimmune diseases [7,8,9]. Microbial transglutaminase is responsible for catalyzing protein cross-linking, which means that it can potentially modify endogenous naïve proteins, making them immunogenic [10].

**Figure 1:**
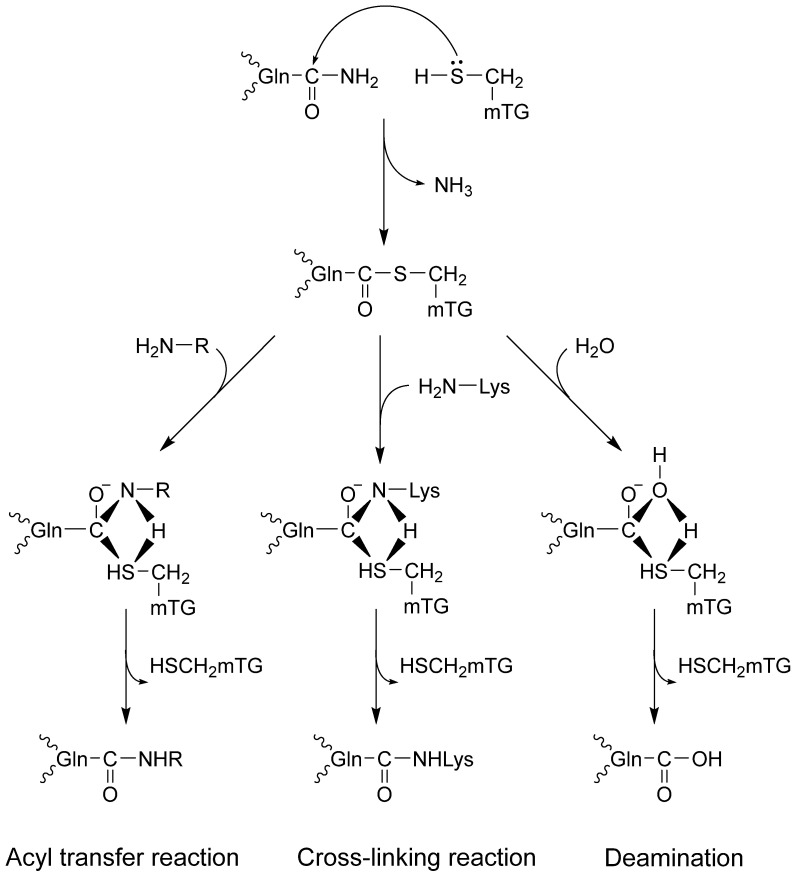
Microbial TG-catalyzed reactions.

Transglutaminases might change proteins by incorporating amines and disturbing intra- and intermolecular cross-links or deamidation, causing profound changes in their molecular structure [10,11,12]. The catalytic triad (Cys, His, Asp) in the active site of the enzyme combines with glutamine in the protein, forming a thioester with γ-carboxamide and releasing ammonia. It is a rising enzyme–protein complex. There are three possible reaction routes with nucleophiles (Figure 1). First, an isopeptide bond may form with the chain protein. The second option is to bind the amine via a γ-glutamyl bond, which enriches the protein with another amino acid. The third route leads to deamination in the reaction with water [1,2,12,13].

In the human body, TG occurs in many isoforms, including TG1 and TG2. TG 2 (Tissue transglutaminase) is a protein with a mass of 80,000 Da. This enzyme is a dimer and consists of four domains (an N-terminal, a catalytic center, and two C-terminal ends). Unlike TG2, mTG does not require the presence of Ca^2+^ ions. The optimal conditions for its activity are 25 ÷ 50 °C and pH in the range of 5.0 ÷ 9.0. These properties greatly facilitate its use on an industrial scale and make this enzyme an attractive technological substrate for the food industry.

Microbial transglutaminase is considered a processing aid and was granted GRAS (Generally Recognized as Safe) status many years ago. As a result, it has not been evaluated for many years and is still not evaluated against current public health toxicity and safety criteria [14]. However, mTG and its transamidated complexes have pro-inflammatory, immunogenic, pathogenic, and potentially toxic effects, and thus they may pose a threat to public health [15,16].

There have been studies suggesting that mTG is capable of cross-reactivity with human mitochondrial M2 antigen, ANA and other nuclear antigens. The work of Lerner et al. [17] confirms the relationship between mTG and the possibility of developing autoimmune diseases. The authors showed that there is a similarity in its amino acid sequence, and therefore cross-reactivity between mTG and various tissue antigens, such as fibrinogen, histone, and creatine kinase S-type, is possible. Sequence similarities between the enzyme and human epitopes, representing targets in autoimmune diseases such as inflammatory bowel diseases and celiac disease—autoimmune diseases of the nervous system—were examined [8,17,18,19,20,21,22,23,24,25,26]. Another paper by Lerner et al. shows that wheat products treated with mTG are immunoreactive in patients with CD [18]. In turn, a review by Lerner & Benzvi discusses the mechanisms and pathways of the gluten–gut–brain axis that link wheat and gluten consumption to neurodegenerative disease, including gluten-induced dysbiosis, increased intestinal and systemic permeability, cross-reactive antibodies, and sequence homology between brain antigens and gluten [25]. Liver disease was also potentially considered [17].

Of course, as mTG belongs to the transglutaminase family and can functionally imitate tissue transglutaminase, it is a potential inducer of celiac disease [9,27].

Recent studies have shown that mTG can bind to tissue transglutaminase (tTG), which is an autoantigen of celiac disease [7,8]. Lerner et al., presenting the method of microbial transglutaminase penetration into human tissues, consider the transcytosis of mTG into the subepithelial compartment or immune reactions against mTG–gliadin complexes, as well as the resistance of mTG to oxidative stress [16,17,21]. Gliadin peptides can be good substrates for transglutaminases—both tTG and mTG [17]. After the formation of cross-linked tTG/mTG gliadin complexes, neoepitopes appear on their surfaces. The presence of neoepitopic tTG (tTG neo) and mTG (mTG-neo) antibodies is detected in the patients’ sera [26]. The gluten part of the complex may be related to intestinal permeability [28]. Tight junction protein sequences have glutaminyl and proline residues that are linked to the peptidyl. Occludins, myosin, zonulin, and keratin may be very good substrates for mTG-mediated cross-linking and thus may cause a decrease in intestinal permeability [17]. Once mTG binds glutamine residues, glutamine deficiency may occur, which in turn may cause increased intestinal permeability [7]. Next, possible pathogenic mechanisms that link mTG to the etiology of CD include the suppression of mechanical and immunological intestinal barriers [20,29], stimulating bacterial growth, and increasing the uptake of gliadin peptide [30]. Gliadin and mTG molecules can be co-transcytosed through enterocytes. The potential uptake of mTG–gliadin complexes by dendritic cells of the mucosa can also be considered [31].

Steffen et al. report the discovery of an MTG from *Kutzneria albida* (KalbTG), which exhibited no cross-reactivity with known MTG substrates or commonly used target proteins, such as antibodies. KalbTG was produced in *Escherichia coli* as a soluble and active enzyme in the presence of its natural inhibitor ammonium, preventing potentially toxic cross-linking activity [32].

Our research focused only on mTG from *Streptoverticillium moboarense*, and its complexes can have various harmful effects [12]. Some reports state that the enzyme is deactivated by heating or acidification of the stomach, and its covalently connected isopeptide bonds are safe. On the other hand, the extended thermostability and wider pH range of mTG activity contradict these arguments.

Primary biliary cholangitis (PBC) is a cholestatic, autoimmune liver disease characterized by the occurrence of anti-mitochondrial (AMA) and antinuclear antibodies in the patients’ sera [33,34,35,36,37,38,39,40]. AMA are found in up to 90% of PBC patients and target components of the 2-oxoacid dehydrogenase complexes, mainly the E2 subunit of the pyruvate dehydrogenase complex (PDC-E2) [41,42]. Autoreactive T cells specific to the dominant autoantigen PDC-E2 mediate the destruction of bile cells specific to this autoantigen. AMA levels are not associated with disease severity and progression. Different AMA IgG subclasses have different clinical implications. PBC patients positive for IgG3 AMA had histologically more progressive disease and were more frequently cirrhotic than those who were negative. AMA may occur due to factors such as oxidative damage, molecular mimicry, and apoptosis of biliary epithelial cells. We can distinguish several nuclear structures that are targets for ANAs in PBC. Some ANAs are specific for nuclear envelope (NE) proteins such as antibodies against the glycoprotein gp210 [43]. ANAs directed against NE proteins, such as anti-p62 antibodies, are not very common but are highly specific for PBC. Anti-gp210 and anti-p62 antibodies are markers of a less favorable prognosis and a more aggressive form of the disease in PBC [44,45]. Patients with a positive anti-p62 antibody test result had higher serum bilirubin levels and more pronounced inflammatory infiltrates according to a liver biopsy. Lamin B receptor is another NE-related protein. In our previous work, we found that anti-LBR antibodies are present in approximately 8–15% of PBC patients [45,46]. The ANA pattern in PBC also takes the form of nuclear dots (MND-ANA). The MND pattern characteristic of PBC is associated with antibodies directed against structural components of the nuclear proteins of promyelocytic leukemia—PML NB such as Sp100, PML, and Sp140. Anti-Sp100, anti-SP140 and anti-PML autoantibodies can be detected in 20–30% of patients with PBC [39,47]. In particular, PBC patients with urinary tract infections were characterized by the presence of antibodies against sp100, which supports the hypothesis that infections such as *Escherichia coli* participate in the stimulation of PBC-specific autoimmunity. The most recently identified biomarkers in PBC are antibodies against kelch-like peptide 12 (KLHL12) and against hexokinase 1 (HK-1) [45,48].

Over the last 20 years, attempts have been made to describe the association between microbiological factors and the etiopathogenesis of the disease [49,50]. The molecular mimicry between human antigens and microbiological proteins may explain the reducing tolerance to specific autoantigens and the development of the disease [51,52,53,54,55].

Our goal was to determine epitope similarity and cross-reactivity between mTG and PBC-specific antigens and to investigate whether the bacterial enzyme may be associated with the induction of autoimmunity due to epitope similarity and cross-reactivity.

## 2. Results

**Figure 2:**
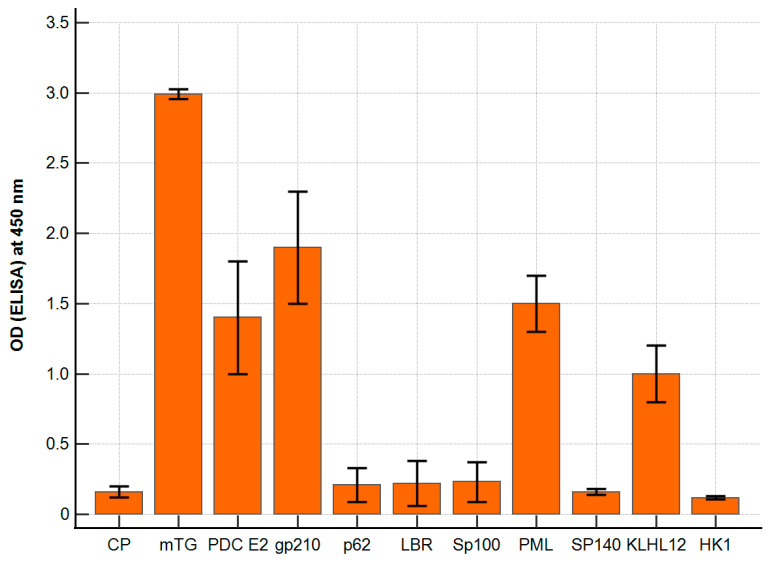
Reaction of anti-mTG rabbit polyclonal antibody to PBC-specific antigens expressed as ELISA ODs.

The polyclonal antibody against mTG reacted with PBC-specific antigens with varying intensities. The ELISA background and a control peptide were considered; our negative control resulted in ODs of around 0.14 ± 0.06 and 0.16 ± 0.02, respectively. The calculated cut-off value (mean value + 2 SD) was 0.20. For antigens p62, LBR, Sp100, Sp140, and HK-1, the absorbance obtained was similar to that of the control peptide, OD = 0.10–0.26, and there were no significant differences. The remaining antigens, PDC E2, PML, and KLHL12, showed significantly higher reactivity: 1.4 ± 0.3, *p* < 0.001; 1.5 ± 0.2, *p* < 0.001; 1.0 ± 0.2; *p* < 0.001. The highest activity was found for the gp210 antigen—OD = 1.95 ± 0.3; *p* < 0.001 (Figure 2).

**Figure 3:**
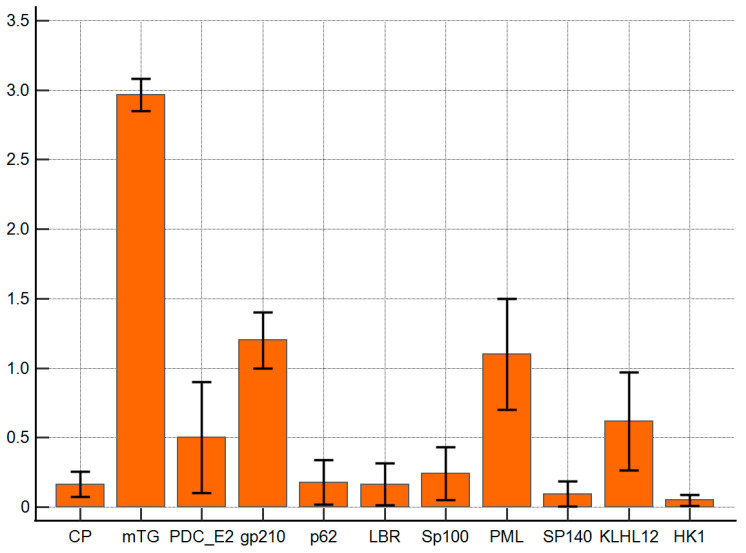
Reaction of anti-mTG mouse monoclonal antibody to PBC-specific antigens expressed as ELISA ODs.

The reaction of anti-mTG mouse monoclonal antibody to PBC-specific antigens expressed as ELISA ODs is presented in Figure 3. The monoclonal antibody against mTG reacted with PBC-specific antigens with lower intensities than polyclonal ones. The ELISA background and control peptide ODs were 0.08 ± 0.03 and 0.16 ± 0.06, respectively, and the cut-off value was 0.22. PDC E2, gp210, PML, and KLHL12 also demonstrated significantly higher reactivity than other complexes: OD = 0.5 ± 0.2, *p* = 0.048; 1.2 ± 0.2, *p* = 0.001; 1.1 ± 0.3 *p* = 0.006, and 0.6 ± 0.2, *p* = 0.022, respectively.

**Figure 4:**
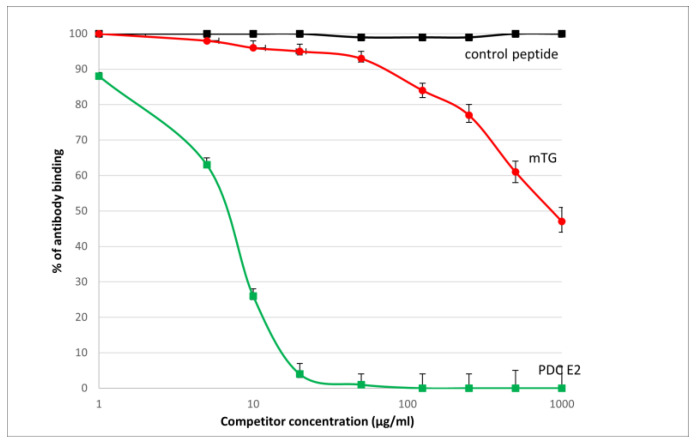
Inhibition of antibody binding to human PDC-E2 by microbial peptide mTG.

**Figure 5:**
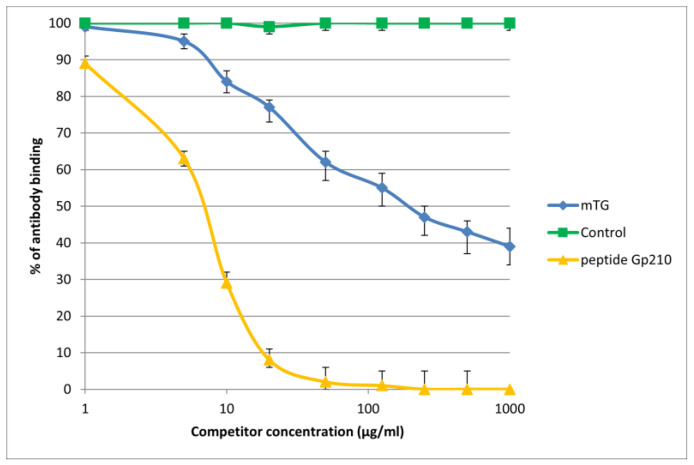
Inhibition of antibody binding to human gp210 by microbial peptide mTG.

**Figure 6:**
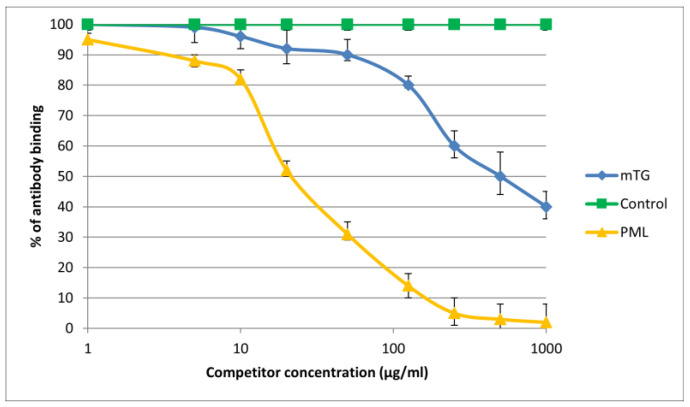
Inhibition of antibody binding to human PML by microbial peptide mTG.

**Figure 7:**
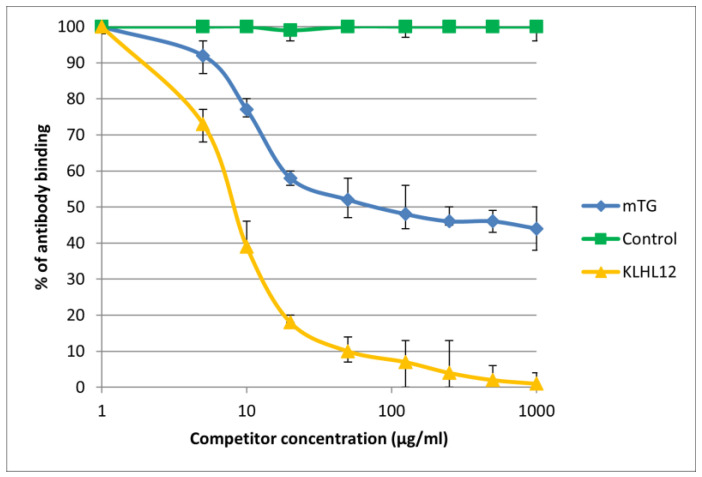
Inhibition of antibody binding to human KLHL12 by microbial peptide mTG.

The reaction of inhibition of specific antigen–antibody binding by mTG was performed for all PBC main antigens. We noticed inhibition of specific antigen–antibody binding by the microbial peptide (mTG) to the PDC M2, gp210, PML, and KLHL12 protein (Figure 4, Figure 5, Figure 6 and Figure 7). In the remaining cases, no significant difference was observed in the inhibition of specific binding between the control peptide and mTG.

The immunological activity of microbial peptide (mTG) expressed as the amount of protein needed to inhibit 50% of the binding of autoantibodies to the human-specific antigen were the best for glycoprotein gp210 and KLHL12 protein 180 µ/mL and 80 µ/mL, respectively.

These PBC-specific peptides corresponded to the region of similarity between bacterial peptide and human PDC-E2 or gp210 or PML or KLHL12: APAASSAGPSFRAP vs. AATASPPTPSAQAP, AGPS vs. AGPS, ASSAG vs. ASGAG, GPSFRA vs. GPRTRA, respectively.

## 3. Discussion

Microbial TG has been found in meats and processed meat products offered in supermarkets, which suggests that mTG may not be totally destroyed during industrial food processing [25].

Consuming industrially processed foods with added mTG may potentially affect the human gastrointestinal tract [53]. Various harmful effects have been attributed to microbial TG and possible pathogenic mechanisms have been discussed [5,6,15,19,23,27,55,56,57].

In recent years, many studies have described the potential role of mTG in the induction of CD and other autoimmune diseases [6,7,14,15,16,17,18,19,20,21,22,23].

Lerner et al. recently described cross-reactivity and sequence similarity between mTG and various human epitopes, highlighting two novel mechanisms for mTG-mediated autoimmunity induction [15]. They performed sequence alignment between mTG and various antigens specific to autoimmune diseases. Monoclonal and polyclonal antibodies prepared specifically against mTG were applied to 77 different human tissue antigens using ‘in-house’ ELISA. The authors noticed that six antigens shared significant homology with mTG immunogenic sequences, representing major targets of common autoimmune conditions. The alignment cut-off was kept at a minimum of seven identical AAs, and a peptide length of ≥12 AAs. In our work, the alignment cut-off was kept at a lower level, at a minimum of four identical AAs. We detected four pairs of similar sequences between human PBC-specific antigens and mTG epitopes. We also performed a similar experiment with both polyclonal and monoclonal antibodies only for PBC-specific proteins. As for PBC-specific antigens, the work of Lerner et al. examined only PDC M2 [16]. In both works, the antibody reaction with the PDC E2 antigen was significantly higher. Lerner et al. found that the reaction of a polyclonal antibody with the mitochondrial M2 antigen gave an OD of 2.79, in our work the highest OD value for this reaction was 1.4. This difference may of course be related to the use of different antigens or the conditions under which the ELISA tests were performed, because in both studies they were not commercial tests. We obtained higher OD values with the polyclonal antibody, which was expected, since it is a heterogeneous mixture of antibodies produced by different B cell plasma clones against different epitopes of the entire antigen. A monoclonal antibody is a homogeneous population of antibodies produced by a single B cell clone. The substrate activity of mTG is much broader than that own tTG, produced inside an organism or cell, which may cause slightly less-specific reactions. The reaction with the monoclonal antibody provided comparable results in both studies. The remaining PBC-specific antigens have not been previously tested for reaction with mTG antibodies. Additionally, we performed the inhibition of specific antigen–antibody binding by mTG to four PBC-specific antigens: PDC M2, gp210, Sp100, and PML. We noticed inhibition of specific antigen–antibody binding to the antigens by this microbial peptide. The immunological activities of microbial peptide (mTG) expressed as the amount of protein needed to inhibit 50% of the binding of autoantibodies to the human-specific antigen were the best for glycoprotein gp210 and KLHL12 protein.

Tissue TG is important for the degradation of injured mitochondria [58]. Tissue transglutaminase not only modulates the apoptosis process itself, but also, by stabilizing the proteins changed during this process, prevents the immune system from being exposed to neoantigens formed during this process. In the event that this mechanism is disrupted, autoimmune diseases may develop. On the one hand, it is believed that insufficient removal of autoreactive lymphatic cells by apoptosis may reduce tolerance towards self-antigens. On the other hand, an excess of cells undergoing apoptosis and insufficient removal by phagocytic cells may also lead to autoimmunity. Many autoantigens have been found in apoptotic bodies, and the presence of tTG has also been found in these bodies. Enzyme activity affects mitochondrial function as well as mitochondrial molecules that are responsible for energy balance. TG may be involved in the degradation of damaged mitochondria. However, it has not yet been thoroughly investigated whether mTG can replace tTG in all cases. Microbial TG, acting as a survival factor, may potentially cause the naïve peptide to transform into an immunogenic one, which could result in impaired immunological tolerance. The dihydrolipoyllysine-residue acetyltransferase component of the pyruvate dehydrogenase complex is an important enzyme in mitochondrial energy metabolism and preservation. The bacterial enzyme can imitate the functions of the human one [59].

Environmental factors-mediated activation of the autoimmune response and subsequent autoimmune diseases, including PBC, could be caused by molecular mimicry, bystander activation, epitope spreading, or by all of these factors [54,60]. Kikuchi et al. incubated peripheral blood mononuclear cells of PBC patients with short non-protein CpG-DNA sequences (unmethylated cytosine–phosphate–guanine—CpG motifs), which are commonly present in the cells of various bacteria. An increase in the number of activated B lymphocytes was found in PBC patients [61]. As a result of these mechanisms, additional autoantigens such as gp210, PML protein, or KLHL12 can be released from the damaged tissue. Gp210 is composed of an amino-terminal domain of 1783 amino acids (located in the perinuclear space), a 20-amino-acid transmembrane segment, and a cytoplasmic carboxyterminal tail domain of 58 amino acids. The carboxyl terminus of gp210, with its domain oriented to the outside of the nuclear lumen and supporting the nuclear pore complex, is thought to be particularly antigenic [62]. Samples collected from patients with PBC show increased expression of the gp210 antigen on the nuclear envelope of biliary epithelial cells (BECs) of the small bile ducts. There is a correlation between the expression level of gp210 in BEC of small bile ducts and inflammation in PBC [63]. Glycoprotein Gp210 plays a key role in nuclear pore biogenesis [64]. The direction of gp210 depends on its transmembrane segment and its C-terminal tail. These domains mediate the oligomerization of interactions with other NUPs [64]. Anti-gp210 antibodies appear; their quantity correlates with an increased IgG concentration, which may be associated with the response to increased bacterial infection. It has also been shown that anti-gp210 antibodies may be associated with a change in the gut microbiome [65]. PML protein act as transcriptional regulator, its function is a wide range of important cellular processes, including tumor suppression, transcriptional regulation, apoptosis, senescence, DNA damage response, and viral defense mechanisms. The kelch-like family of proteins is involved in many cellular functions, including cell structure, cellular communication, transcriptional regulation, collagen export, and ubiquitination of proteins. KLHL12, which is placed inside the nucleus, is crucial for collagen export [66,67]. In our work, we show for the first time a possible association of mTG with PBC and the possibility of molecular mimicry between the environmental enzyme and endogenous human autoantigens.

There may also be a connection between celiac disease and PBC. Immune disorders affecting the small intestine and liver are associated with an imbalance of the intestinal–hepatic axis [68]. Once the cross-linked complexes enter the intestinal tract, they remain undigested and have the potential to cross epithelial barriers, thus encountering antigen-presenting cells. Due to increased intestinal permeability, molecules resulting from the cross-linking of tissue transglutaminase with bacterial antigens, such as mTG, enter the liver through the portal circulation. Molecular mimicry between bacterial antigens and the pyruvate dehydrogenase component E2, gp210, PML, or KLHL12 recognized by autoantibodies plays a role in the pathogenesis of PBC. We are dealing with incorrect guidance of intestinal T lymphocytes to the liver, which contributes to its immunological damage.

Some works have suggested the importance of mTgs for the survival of microorganisms. It exerts anti-protease activity, suppresses anti-microbial peptides, has emulsifying activity, is anti-phagocytic, and affects Th1/Th2 balance [23]. Autoimmune diseases including PBC are associated with intestinal dysbiosis. Microbial TG forms isopeptide bonds that cannot be hydrolyzed by any known eukaryotic enzyme. They are also resistant to reducing agents, detergents, and chaotropic agents. Once in the enteric lumen, these enzyme-docked peptide complexes persist without being digested or destroyed [14,23,69,70]. This allows the microorganisms to survive in the gut for much longer, exhibiting resistance to local peptidases, bile acids in the gut lumen, and pH fluctuations. The human intestinal microflora plays a key role in human health, and is involved in many functions, including nutritional, metabolic, signaling, developmental, and immune processes occurring in the human body [54].

When used in the food industry, mTG causes higher enzymatic activity in the intestines, which may lead to dysbiosis and pathobiosis. The presence of a pathobiome is one of the main risk factors for more severe pathologies. It appears that both nutrients and processed food additives can affect the gut ecosystem and may also be associated with disruption of tight junction integrity [71]. When the intestine becomes leaky, immunogenic molecules can enter the circulation, causing, e.g., PBC. Zonulin, claudins, F-actin, and occludins are very important for the proper functioning of tight junctions. At the same time, they are also very good substrates for mTG because they contain acyl donors and acyl acceptors [72]. Transamidation with mTG can cause the leakage of tight junctions in the intestine. One of the characteristic features of mTG is its emulsifying effect, and emulsifiers disrupt the function of the intestinal tight junctions [73]. Since a leaky gut/liver are linked, it seems that these factors may affect liver activity and may be involved in autoimmune liver diseases as well.

Researchers’ opinions on the use of mTG in the food industry are divergent. Some scientists call for a reassessment of the safety of enzymatic applications in food processing and the use of mTG [5,30,74,75]. Whether mTG plays a direct role in stimulating autoimmune diseases is still a matter of debate at this point. However, reports indicating that the bacterial enzyme can penetrate the intestinal epithelial lining and cause autoimmune disease would require further study regarding the potential threat, even if it concerns a small population.

## 4. Materials and Methods

### 4.1. Microbial Transglutaminase—mTG (Zedira GmbH, Darmstadt, Germany)

mTG is recombinantly produced in *E. coli.* A gene derived from *Streptomyces mobaraensis* was used. Microbial transglutaminase is purified by a series of column chromatography steps. The final quality control is summarized in Table 1.

### 4.2. Cross-Reactivity Between mTG and PBC-Specific Antigens Using a Polyclonal Antibody for Recombinant mTG

Cross-reactivity between mTG- and PBC-specific antigens was demonstrated using ‘in house’ ELISA technique. Rabbit polyclonal antibodies associated with recombinant mTG (Zedira GmbH, Darmstadt, Germany) were applied to nine different antigens specific to PBC and one control antigen non-specific to PBC using the ELISA method. The ELISA method was extracted from various manuscripts published by Lerner et al. [17] and Vojdani et al. [76.77] and applied with some modifications. Different wells of ELISA plates were coated with various antigens: PDC-E2, gp-210, p62, LBR, Sp100, Sp140, PML, KLHL12, HK-1, and a control peptide (a peptide that is not a specific antigen in autoimmune diseases) as a negative control. Each antigen was dissolved in 0.01 M carbonate buffer pH 9.6, in optimal amounts ranging from 2.5 µg/mL to 1.0 µg/mL, and was added to triplet wells. Following incubation for 24 h at room temperature, the plates were washed 5 times, after which 200 microliters of blocker containing 5% bovine serum albumin was added to each well. After incubation (24 h in 4 °C) and washing, rabbit polyclonal mTG was added at a dilution of 1:300. Plates were incubated for 1 h at room temperature, and after additional washing, 100 microliters of peroxidase-conjugated anti-rabbit mouse IgG (Daco A/S, Hvidovre, Denmark, dilution 1:2000) was added to different sets of ELISA plates. After the incubation and washing were repeated again, 100 microliters of substrate—TMB was added to each well, and color development was measured at 450 nm.

### 4.3. Cross-Reactivity Between mTG and PBC-Specific Antigens Using Monoclonal Antibody to Recombinant mTG

Cross-reactivity between mTG and PBC-specific antigens was demonstrated using ‘in house’ ELISA. Mouse monoclonal antibodies related to recombinant mTG (Zedira GmbH, Darmstadt, Germany) were applied to nine different antigens specific to PBC and one control antigen non-specific to PBC using the ELISA method. The ELISA method used was extracted from various manuscripts published by Lerner et al. [17] and Vojdani et al. [76,77] and applied with some modifications. Different wells of ELISA plates were coated with various antigens: PDC-E2, gp-210, p62, LBR, Sp100, Sp140, PML, KLHL12, HK-1, and a control peptide. Each antigen was dissolved in 0.01 M carbonate buffer at pH 9.6, in optimal amounts ranging from 2.5 µg/mL to 1.0 µg/mL, and was added to triplet wells. Following incubation for 24 h at room temperature, the plates were washed 5 times, after which 200 microliters of blocker containing 5% bovine serum albumin was added to each well. After incubation (24 h in 4 °C) and washing, mouse monoclonal antibody against mTG was added at a dilution of 1:150. Plates were incubated for 1 h at room temperature, and after another wash, 100 microliters of peroxidase-conjugated anti-mouse rabbit IgG (Daco A/S, Hvidovre, Denmark, dilution 1:2000) was added to different sets of ELISA plates. After incubation and washing were repeated again, 100 microliters of substrate—TMB—was added to each well, and color development was measured at 450 nm.

### 4.4. Detection of Inhibition of Specific Antibody–Antigen Binding

#### 4.4.1. Synthetic and Recombinant Peptides Used in the Study

Recombinant microbial transglutaminase, mTG (Zedira GmbH, Darmstadt, Germany), human recombinant dihydrolipoyllysine-residue acetyltransferase component of pyruvate dehydrogenase complex, mitochondrial–PDC E-2 (Biomatik USA, LLC, Wilmington, NC, USA), human recombinant gp-210 protein (Abnova, Taipei, Taiwan), human recombinant PML protein (Abnova, Taipei, Taiwan), human recombinant KLHL12 protein (Abnova, Taipei, Taiwan), and a control peptide (Yale University, New Haven, CT, USA) were used in this study.

#### 4.4.2. Sera of Patients Used in the Study

The study group was composed of 92 Polish patients with PBC (87 women and 5 men; median age 51, range 26–74 years), who were diagnosed over the past 10 years. The diagnosis was based on internationally accepted criteria [78]. The sera of patients positive for the hepatitis B surface antigen (HBsAg), anti-hepatitis A (IgM), and hepatitis C virus were excluded, and we also excluded patients with alcoholism and AIH (autoimmune hepatitis)/PBC overlap syndrome. We also excluded patients with other autoimmune diseases. The control group contained serum samples from 30 healthy adult blood donors. The study procedure was conducted according to the ethical guidelines of the Declaration of Helsinki and was accepted by the Centre of Postgraduate Medical Education’s ethical committee (Warsaw; approval number 71/PB/2019). Sera were selected according to their antibody activities, as determined by their corresponding ELISAs.

#### 4.4.3. Procedure of Detection of Inhibition of Specific Antibody–Antigen Binding

We detected the specific inhibition of antibody–antigen binding by the in-house ELISA method. To investigate whether concurrent reactivity to mTG was due to cross-reactivity, a competitive ELISA was performed by measuring the reactivity of anti-PDC E-2, anti-gp210, anti-PML, and anti-KLHL12 antibodies present in human serum after exposure to mTG and control peptides as liquid phase competitors (final concentrations: 0, 5, 10, 50, 500, and 1000 µg/mL). All experiments were performed in 96-well antigen-coated microplates (maxi-sorp, Nunc, Roskilde, Denmark). Non-specific absorption was prevented by the addition of 200 mL/well of 5% bovine serum albumin (BSA) in phosphate-buffered saline (PBS) and plates were incubated at 4 °C overnight.

Plates (maxi-sorp, Nunc, Roskilde, Denmark) were coated overnight with suitable concentrations of the peptides in 0.1 M bicarbonate buffer pH 9.6, then saturated with 5% bovine serum albumin in PBS and washed. The mTG in consecutive dilutions were incubated for 2 h with shaking, with PBC patients’ sera containing a specific antibody at a constant dilution (1:100). After equilibrium had been reached, 100 mL of each solution was transferred to an ELISA plate coated with the antigen, incubated for 1 h, and washed. Next, the plates were washed, incubated for 1 h at room temperature with peroxidase-conjugated anti-human rabbit IgG (Daco A/S, Hvidovre, Denmark, dilution 1:3000), and washed once again. The color reaction was developed by adding tetramethylbenzidine (TMB, Serva, Heidelberg, Germany) ) for 15 min and stopping the reaction with 1N H_2_SO_4_. The optical density (OD) was measured at 450 nm.

## Figures and Tables

**Table 1:** Final quality control for mTG according to the manufacturers’ instructions.

Parameter	Specification
Protein concentration	10 mg/mL
Activity	>270 U/mL
Protein mass	383.3 kDa
Purity according to AEX-HPLC	>98%
SDS-PAGE	Single band at ~38 kDa
Host cell protein content	<0.15 ng/U
Host cell DNA content	<0.12 ng/U
Endotoxin content	<0.004 EU/U
Sterility	No growth

## Data Availability

The raw data supporting the article conclusions will be made available by the authors, without undue reservation.
